# Endophyte-Mediated Resistance in Tomato to *Fusarium oxysporum* Is Independent of ET, JA, and SA

**DOI:** 10.3389/fpls.2019.00979

**Published:** 2019-07-31

**Authors:** Maria E. Constantin, Francisco J. de Lamo, Babette V. Vlieger, Martijn Rep, Frank L. W. Takken

**Affiliations:** Molecular Plant Pathology, Faculty of Science, Swammerdam Institute for Life Sciences, University of Amsterdam, Amsterdam, Netherlands

**Keywords:** systemic acquired resistance (SAR), induced systemic resistance (ISR), Fusarium wilt, root pathogens, vasculature, biocontrol, Fo47, phytohormones

## Abstract

Root endophytes can confer resistance against plant pathogens by direct antagonism or via the host by triggering induced resistance. The latter response typically relies on jasmonic acid (JA)/ethylene (ET)-depended signaling pathways, but can also be triggered via salicylic acid (SA)-dependent signaling pathways. Here, we set out to determine if endophyte-mediated resistance (EMR), conferred by the Fusarium endophyte Fo47, against Fusarium wilt disease in tomato is mediated via SA, ET or JA. To test the contribution of SA, ET, and JA in EMR we performed bioassays with Fo47 and *Fusarium oxysporum* f. sp. *lycopersici* in tomato plants impaired in SA accumulation (*NahG*), JA biosynthesis (*def1*) or ET-production (*ACD*) and -sensing (*Nr)*. We observed that the colonization pattern of Fo47 in stems of wildtype plants was indistinguishable from that of the hormone mutants. Surprisingly, EMR was not compromised in the lines affected in JA, ET, or SA signaling. The independence of EMR on SA, JA, and ET implies that this induced resistance-response against Fusarium wilt disease is distinct from the classical Induced Systemic Resistance (ISR) response, providing exciting possibilities for control of wilt diseases independent of conventional defense pathways.

## Introduction

Endophytes are microorganisms that can inhabit plant tissues without causing disease. Notably, there are many examples of endophytes that decrease disease susceptibility of their host upon pathogen infection. This property makes endophytes attractive agents for organic farming. How endophytes succeed in suppressing disease symptom severity depends on the fungal strain and the host plant being studied (Busby et al., 2016). Two general modes of action have been proposed: direct and indirect antagonism. The first term is used for endophytic strains that have a direct effect against the pathogen by processes such as parasitism, antibiosis or competition for resources or infection sites (Fravel et al., 2003). Indirect antagonism describes an effect of the endophyte on the pathogen that is mediated via the host plant, for instance by triggering an Induced Systemic Resistance (ISR) response in the latter. ISR is normally triggered upon endophytic colonization of the roots, and primes the plant immune system for resistance against future pathogen attacks. Endophytes trigger ISR via the phytohormones jasmonic acid (JA) and ethylene (ET), resulting in a faster and stronger immune response following pathogen attack. Some endophytes are able to trigger a Systemic Acquired Resistance response (SAR) that is salicylic-acid (SA) dependent, and also results in primed host defenses (Sequeira, 1983; Fu and Dong, 2013; Pieterse et al., 2014).

Disease-suppressive soils often contain beneficial microorganisms that upon colonization of a plant confer protection against pathogen attack (Pieterse et al., 2014). One example of such a microorganism is the fungal endophyte *Fusarium oxysporum* strain Fo47. This strain was originally isolated from melon wilt-disease suppressive soils (Alabouvette, 1986), and Fo47 has later been found to confer Endophyte-Mediated Resistance (EMR) in various crop species. Fo47 is able to confer resistance against pathogenic Fusarium wilt of carnation (Lemanceau et al., 1993), flax (Duijff et al., 1998; Trouvelot et al., 2002), asparagus (Elmer, 2004), watermelon (Larkin and Fravel, 1999), and tomato (Lemanceau and Alabouvette, 1991; Fuchs et al., 1997, 1999; Larkin and Fravel, 1999; de Lamo et al., 2018). In addition, Fo47 has been shown to reduce susceptibly in pepper against Verticillium wilt (Veloso and Diaz, 2012; Veloso et al., 2016). Furthermore, in cucumber, Fo47 was found to confer EMR against the root-rot pathogen *Pythium ultimum –* an oomycete – which causes damping-off disease (Benhamou et al., 2002). In pepper, Fo47 can confer protection against the root-rot and blight causing pathogen *Phytophthora capsici*, but only when the pathogen was present in the soil (Veloso and Diaz, 2012). When *Phytophthora* was applied on the leaves, Fo47-mediated protection was very weak and disappeared three days after inoculation. Notably, Fo47 does not confer protection against leaf-infecting pathogens, such as *Botrytis cinerea* (Veloso and Diaz, 2012). It has been proposed that Fo47 confers EMR in tomato via various mechanisms, such as direct antagonism (by competition) and ISR (Fravel et al., 2003; Aimé et al., 2013). The broad-spectrum resistance conferred by this endophytic strain makes it an attractive model for research and for potential application.

*F. oxysporum* and *Verticillium dahliae* are known for their ability to cause wilt disease in a wide variety of plant species (Talboys, 1972). The hyphae of these vascular fungi can enter the root by penetrating directly through the cortical root cells or via wounds (Talboys, 1972). After invasion, the fungal mycelium advances intercellularly and intracellularly through the root cortex until it reaches the xylem vessels and subsequently colonizes these through the pits (Bishop and Cooper, 1983; Beckman and Roberts, 1995; Michielse and Rep, 2009). Once in the vasculature the fungus proliferates and spreads. In an attempt to limit vessel colonization the host induces different defense responses, which include the production of gels, gums, and tyloses (Di Pietro et al., 2003). These compounds block the vessels, thereby compromising the plant’s ability to transport water, giving rise to the typical wilting symptoms. Another mechanism by which plants attempt to limit root colonization is by producing papillae at penetration sites (Yadeta and Thomma, 2013). Whereas in pea roots this response is strongly induced by the endophyte Fo47 it is suppressed following *Fo* f. sp. *pisi* inoculation (Benhamou and Garand, 2001).

Fusarium wilt disease in tomato is caused by *F. oxysporum* f. sp. *lycopersici* (Fol). It has been established that plant phytohormones play an important role in disease development in the Fol-tomato interaction (Di et al., 2017). Tomato plants unable to produce ET (*ACD* overexpressing) or sense this hormone (*Nr* mutant), developed fewer disease symptoms upon inoculation with Fol than wild-type plants. In contrast, tomato lines compromised in their ability to accumulate SA (*NahG*) are hyper-susceptible to Fol (Di et al., 2017). JA did not seem to be involved in this interaction, as *def1* mutants that are impaired in biosynthesis of JA showed no difference in disease development following Fol inoculation as compared to the mock treatment (Di et al., 2017). In addition, Fol was observed to colonize *ACD* and *Nr* tomato stems to a lesser extent, while it was able to colonize *NahG* plants more extensively than wild-type plants (Di et al., 2017). Currently, it is unknown whether phytohormones influence colonization of tomato stems by Fo47. Furthermore, it is an open question whether phytohormones are necessary to mount EMR in tomato, and whether the induced resistance response can be classified as ISR or SAR. By using tomato lines impaired in production or perception of ET, JA, or SA, we assess the dependence of these phytohormones for EMR and colonization of the stem by Fo47.

## Materials and Methods

### Plant Growth and Fusarium Culture Conditions

Eight different tomato (*Solanum lycopersicum*) genotypes were used in this study. Four of these are wild-type cultivars: Moneymaker, Castlemart, UC82B, and Pearson. The *NahG* line is a Moneymaker background and constitutively expresses a *NahG* salicylate hydroxylase transgene impairing SA accumulation (Brading et al., 2000). The JA biosynthesis *def1* is in a Castlemart background (Howe et al., 1996). The ACD line, in a UC82B background, overexpresses a gene encoding a ACC deaminase resulting in compromised ET accumulation (Klee et al., 1991), while the dominant *Nr* (Lanahan et al., 1994) mutation in the Pearson background impairs ET perception. The tomato variety C32, which is Moneymaker like, was used for qPCR experiments and microscopy. Plants were grown in a climate-controlled greenhouse with day-night temperature of 25°C, 16 h light/8 h dark regime and a relative humidity of 65%.

### Fusarium Inoculation Assay

Wild-type or transgenic endophytic (Fo47) and pathogenic (Fol4287, race 2) Fusarium strains were used for bioassays (Lemanceau and Alabouvette, 1991; Di Pietro et al., 2003; Ma et al., 2010; Vlaardingerbroek et al., 2015). To facilitate isolation of fusarium from infected stems, a transgenic Fo47 (sFP1544) strain carrying a hygromycin resistance marker (Ma et al., 2010) and a transgenic Fol4287 strain (sFP3059) carrying hygromycin and zeocine resistance markers were used (Vlaardingerbroek et al., 2015). GFP labeled Fo47 (sFP1544) and RFP labeled Fol4287 (sFP1173) (van der Does et al., 2008) or RFP labeled Fo47 (sFP3092) (Vlaardingerbroek et al., 2015) and GFP labeled Fol007 (sFP1079) (van der Does et al., 2008) strains were used for callose quantification experiments. Fungal strains were grown on Potato Dextrose Agar (PDA) plates at 25°C for 7–10 days in the dark. In order to obtain spores a piece of agar from these PDA plates was transferred to 100 mL minimal media (1% KNO_3_, 3% sucrose and 0.17% Yeast Nitrogen Base without amino acids and ammonia), and incubated for 3–5 days at 25°C, 150 rpm. Spores were filtered through a Miracloth filter (Millipore), washed once with sterile water and diluted to a concentration of 10^7^ spores/mL. Co-inoculation treatment of endophytic and pathogenic Fusarium strains consisted of premixing each strain in a ratio of 1:1 (10^7^:10^7^ spores/mL) to produce the inoculum. Ten days old tomato seedlings were uprooted, roots were trimmed to facilitate Fusarium infection and dipped in spore suspension for 5 min and subsequently the seedlings were re-potted in soil. Plant weight and disease symptoms were assessed 3 weeks after inoculation. Disease index (DI) scoring was based on the extent of vessel browning and external symptoms such as yellowing as described (Gawehns et al., 2014), with the addition of a disease index 5 for dead plants. In short, disease index 0 = no symptoms; 1 = brown vessel above the soil; 2 = one or two brown vessels at the cotyledon level; 3 = at least three brown vessels and shows growth distortion, 4 = all vessels brown or the plant is small and wilted, 5 = plant is dead. Each bioassay was repeated at least twice with a minimum of eight plants analyzed each time.

### Fungal Recovery Assay

After disease scoring, tomato stems were collected and surface-sterilized as described (de Lamo et al., 2018). In brief, stem pieces were treated with 70% ethanol and rinsed twice with sterile water under sterile conditions. After surface sterilization, the uttermost parts of the stems were trimmed and two sections of the stem at the cotyledon and the basal part were dissected and placed on PDA plates containing 200 mg/L streptomycin and 100 mg/L penicillin to prevent bacterial growth. In order to specifically allow growth of Fo47, a hygromycin resistant Fo47 strain was used and the PDA plates were supplemented with 100 mg/L hygromycin. Plates were scanned 4 days after incubation in the dark at 25°C.

### Analysis of Gene Expression by Using RT-qPCR

Stem pieces of 3 weeks old tomato plants were ground in liquid nitrogen. RNA was extracted from approximately 200 mg of plant material using Trizol-Reagent (Invitrogen, Life Technologies, Grand Island, NY, United States) according to the manufacturer’s instruction. The RNA was purified afterward with a RNeasy Mini kit (Qiagen, Düsseldorf, Germany) and DNA was removed by on-column treatment with RNase-free DNase (Qiagen). RNA quality was checked by agarose gel electrophoresis and spectrophotometrically. Only RNA samples with an absorbance ratio (260 nm/280 nm) greater than 1.8 were used for cDNA synthesis. First stranded cDNA was synthesized from 1μg of total RNA using the M-MulV reverse-transcriptase RNase H minus kit (Fermentas, Thermo Fisher Scientific, Pittsburgh, PA, United States) according to the manufacturer’s instruction. qPCRs were performed in QuantStudioTM3 (Thermo Fisher Scientific), using the 5× HOT FirePolEvaGreen qPCR Mix Plus (Solis BioDyne). The 10μL PCR contained 10 pM of each primer and 1uL of cDNA diluted 5 times. The cycling program was set to 15 min at 95°C, 40 cycles of 15 s at 95°C, 20 s at 60°C, 30 s at 72°C, followed a melting curve analysis of 15 s at 95°C, 1 min at 60°C, 15 s at 95°C. The gene expression levels were normalized to tomato α-tubulin expression. The primer sequences for gene expression analysis have been described previously by Di et al. (2017), and are listed in Supplementary Table S1. Five biological replicates per treatment with three technical replicates were performed for each gene. The expression levels were calculated using the 2^–ΔCT^ method, using the qbase+ v3.1 program.

### Callose Staining

Ten days old tomato seedlings were incubated either for 5 min or for 3–4 days in either a fusarium spore suspension (10^7^ spores/mL) or in tap water. Afterward, the basal half of the tomato seedlings consisting of the roots and part of the hypocotyl was incubated for 1 h in 0.01% aniline blue solution in PBS. Fusarium colonization and callose were visualized using an EVOS Fl digital inverted microscope equipped with transmitted light, Texas Red (585/29 to 628/32), GFP (470/22 to 510/42 nm), and DAPI (357/44 to 447/60 nm) light cubes. The same settings were used for all image recordings. At least three pictures per plant (minimum of four plants) were analyzed. The FIJI software package from ImageJ v2 was used for image analysis. The captured color images were transformed into 8-bit grayscale pictures. The threshold for each picture was adjusted manually and dark outliers with a radius smaller than 1.0 pixel were removed. The number of callose dots was quantified using the Fiji package. Root surface area was measured by manually selecting the root boundaries using the polygon selection tool and subsequent quantification using the Fiji software (pixels/cm^2^).

### Statistical Analyses

The data on plant fresh weight, disease index and fungal colonization was analyzed using PRISM 7.0. (GraphPad)^1^ by performing a Mann–Whitney *U*-test. For quantifying colonization, we used the following score: 0 = no fungal outgrowth from the stem piece was observed, 1 = fungal outgrowth at crown or cotyledon level, 2 = fungal outgrowth at both crown and cotyledon level. Gene expression was analyzed using a one-way ANOVA test, and by adjusting the *p*-value with a Tukey’s multiple comparisons test. Significant differences of callose deposition among treatments were analyzed by performing a one-way ANOVA, and by adjusting the *p*-value with a Tukey’s multiple comparisons test using PRISM 7.0.

## Results

### SA, JA, and ET Marker Genes Are Not Differentially Expressed Following Endophytic Colonization or Upon Co-inoculation Treatment

**FIGURE 1:**
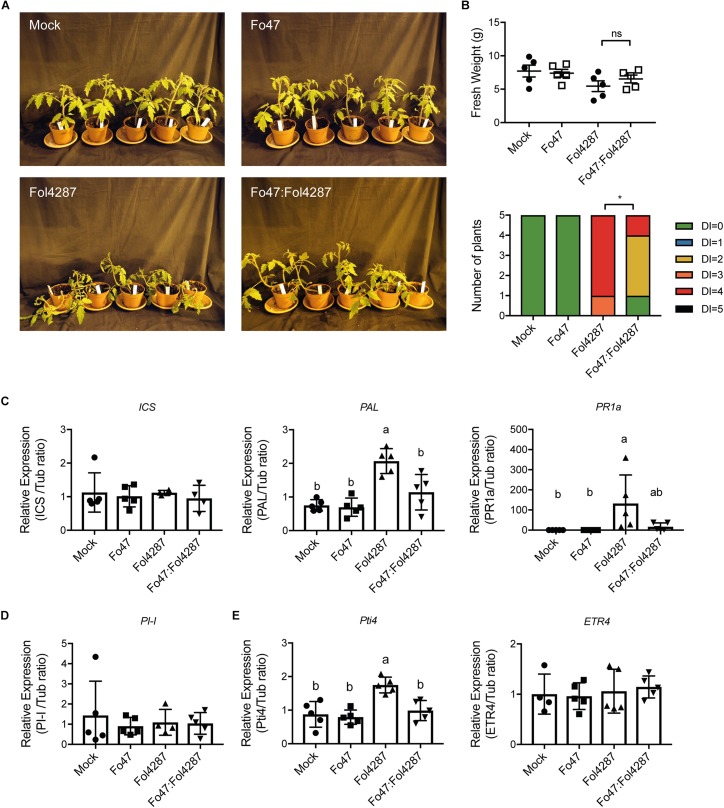
Expression of SA, JA, or ET marker genes is unaffected by EMR. **(A)** Ten days old wild-type tomato seedlings (C32) inoculated with water (mock), Fo47, Fol4287 or co-inoculated with Fo47 and Fol4287. **(B)** Disease development as assessed by measuring fresh weight and disease index 3 weeks after inoculation. Raw data was analyzed by a non-parametric Mann–Whitney *U*-test (^*ns*^*P* > 0.05, ^*^P < 0.05). **(C)** Expression levels of the SA biosynthesis genes *isochorismate synthase* (*ICS*) and *phenylalanine ammonia-lyase* (*PAL*) or the *pathogenesis-related 1a* (*PR1a*) SA maker gene in Fo47, Fol4287 or in co-inoculated plants. **(D)** Expression levels of the JA reporter gene *proteinase inhibitor* (*PI-I*) in Fo47, Fol4287 or in co-inoculated plants. **(E)** Expression levels of the ET-regulated marker genes *ethylene responsive factor* (*Pti4*) and *ethylene receptor* (*ETR4*) in Fo47, Fol4287 or in co-inoculated plants. Gene expression levels were measured using RT-qPCR and depicted relative to that of *tubulin*. Five biological replicates per each treatment were analyzed. The different letters represent a significant difference at *P* < 0.05 as determined by ordinary one-way ANOVA with Tukey’s multiple comparisons test. A repetition of this experiment is shown in Supplementary Material.

To determine the potential involvement of SA, JA, and ET in EMR, the expression of the respective marker genes was analyzed in tomato plants inoculated with water (mock), Fo47, Fol4287 or with both fungal strains. Three weeks after inoculation disease symptoms of the inoculated plants were assessed. After disease scoring the stem section underneath the cotyledons was harvested and RNA was extracted. Subsequent cDNA synthesis enabled measuring expression levels of hormone marker genes via RT-qPCR. Despite having severe disease symptoms (Figures 1A,B and Supplementary Figure S1A), Fol4287-inoculated tomato plants did not show a difference in terms of fresh weight as compared with the co-inoculation treatment (Figure 1B and Supplementary Figure S1B).

To assess whether expression of SA biosynthesis genes was affected upon plant inoculation the expression of two SA biosynthetic genes *Isochorismate synthase* (*ICS*) and *Phenylalanine ammonia-lyase* (*PAL*) was monitored. In addition, the expression of the *Pathogenesis-related 1a* (*PR1a*), a gene previously described to be a reporter for SA-dependent immune signaling, was measured (Kunkel and Brooks, 2002). Fo47 inoculation did not affect expression of *ICS*, *PAL*, or *PR1a* (Figure 1C). Plants infected with Fol4287 did also not show a significant induction of *ICS*, but *PAL* and *PR1a* were induced 3 weeks after inoculation (Figure 1C). Notably, in a second bioassay the induction of *PAL* was not significant upon Fol4287 inoculation, whereas *PR1a* expression was (Supplementary Figure S1C). Upon co-inoculation, *ICS*, *PAL*, and *PR1a* levels remained largely unaffected (Figure 1C), which coincides with the lack of disease symptoms in these plants (Figures 1A,B and Supplementary Figure S1A) (in the second experiment, *PAL* was found to be modestly induced upon co-inoculation (Supplementary Figure S1C)). Taken together, these data indicate that SA responses are not strongly induced upon endophytic colonization and are not consistently upregulated upon co-inoculation; only infection with the pathogen alone consistently induces expression of SA marker genes.

To examine whether JA signaling could be involved in EMR, expression of *Proteinase inhibitor 1* (*PI-I*) was determined. *PI-I* is a reporter for JA-dependent signaling (Mcgurl et al., 1994), and has been used before to monitor JA signaling in tomato stems upon Fol007 inoculation (Di et al., 2017). In Figure 1D, it can be seen that *PI-I* expression was not affected by single inoculations of Fo47 or Fol4287 nor by co-inoculation with both Fusarium strains. These data imply that in co-inoculated plants JA responses are not changed, suggesting that EMR does not involve JA.

To assess the potential role of ET in EMR the expression of two ET response markers, ethylene response factor *Pti4* and the *ethylene receptor gene* (*ETR4*), was analyzed. Upon Fo47 treatment *Pti4* and *ETR4* were not up-regulated (Figure 1E). In Fol4287-inoculated tomato plants, *Pti4* was found to be induced two-fold (Figure 1E) while *ETR4* expression levels remained unchanged (Figure 1E). However, induced expression of *Pti4* upon Fol4287 inoculation could not be reproduced in a second experiment (Supplementary Figure S1E). Upon co-inoculation *Pti4* was not differently expressed in the first bioassay (Supplementary Figure S1E) but appeared repressed in the second bioassay (Supplementary Figure S1E). *ETR4* expression was not affected (Figure 1E), however, in the second bioassay it appeared to be slightly induced (Supplementary Figure S1E). No detectable changes in ET-response genes were observed upon Fo47 inoculation. Taken together, no consistent pattern for induction of ET-related gene expression in EMR was observed, which implies that this hormone does not play a key role in this process.

### EMR Is Independent of SA Accumulation

**FIGURE 2:**
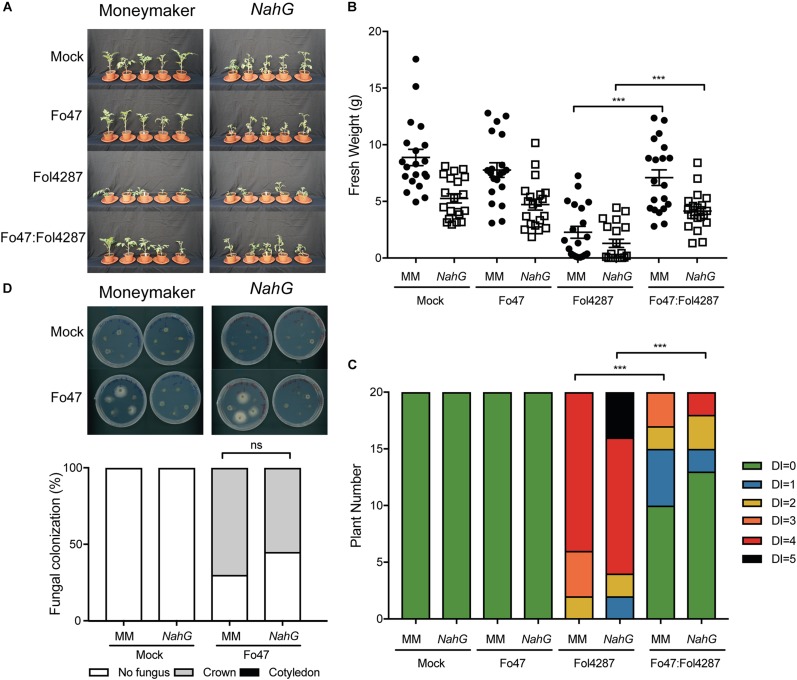
Impaired SA signaling does not affect EMR triggered by Fo47. **(A)** Ten days old tomato seedlings (Moneymaker) and transgenic *NahG* plants impaired in SA accumulation were inoculated with water (mock), Fo47, Fol4287 or co-inoculated with Fo47 and Fol4287. Disease development was assessed by measuring fresh weight **(B)** and disease index **(C)** 3 weeks after inoculation. Raw data was analyzed by a non-parametric Mann–Whitney *U*-test (^∗∗∗^*P* < 0.001). The bioassay was performed three times with similar results, where the number of plants (n) was *n* = 10 for the first repetition and *n* = 20 for the second and third repetition. **(D)** Representative stem pieces harvested from crown (left) and cotyledon (right) level of five individual plants incubated for 4 days on PDA plates. Percentage of Fo47 colonization on mock-inoculated plants (*n* = 10) and Fo47-treated plants (*n* = 20). The experiment was performed three times with similar results. Raw data was analyzed by a non-parametric Mann–Whitney *U*-test (^∗∗∗^*P* < 0.001).

Fo47-mediated resistance has been proposed to be dependent on SA (Aimé et al., 2013), although we did not observe induced expression of the SA marker gene *PR1a* upon co-inoculation of Fo47 and Fol4287 (Figure 2A). If SA is necessary for EMR, then this resistance should not occur in tomato plants unable to accumulate SA. To test this hypothesis, we used a tomato transgenic line containing *NahG*, which encodes a salicylate hydroxylase that converts SA into catechol (Brading et al., 2000). Fusarium bioassays were performed on Moneymaker and *NahG* tomato seedlings by inoculating them with water (mock), endophytic strain Fo47, pathogenic strain Fol4287 or with Fo47 and Fol4287 (in a 1:1 ratio). Three weeks after inoculation, disease symptoms and fresh weight were assessed for both plant genotypes. Moneymaker and *NahG* plants inoculated with Fol4287 developed severe disease symptoms associated with Fusarium wilt, such as leaf epinasty, stunting and leaf yellowing (Figure 2A). Inoculation with the endophytic strain, or co-inoculation with both the endophytic and pathogenic strain, result in no or few external disease symptoms on both tomato genotypes as compared to Fol4287 inoculated plants (Figure 2A). In agreement, co-inoculated plants have a significantly higher fresh weight (Figure 2B) and show significantly less disease symptoms (Figure 2C) than plants inoculated with Fol4287. Therefore, we conclude that SA is not involved in triggering EMR. We then considered that endophytic colonization could be influenced by SA. To test this, Fo47 was re-isolated from tomato stems from Fo47-inoculated plants. Fo47 was identified in Moneymaker and *NahG* tomato stems at the crown level but very rarely at the cotyledon level (Figure 2D). No statistically significant difference in endophytic colonization of *NahG* stems as compared with Moneymaker was observed. Taken together, these data suggest that SA accumulation does not play a major role in triggering EMR nor in colonization by endophytic Fusarium.

### JA Is Not Involved in EMR

**FIGURE 3:**
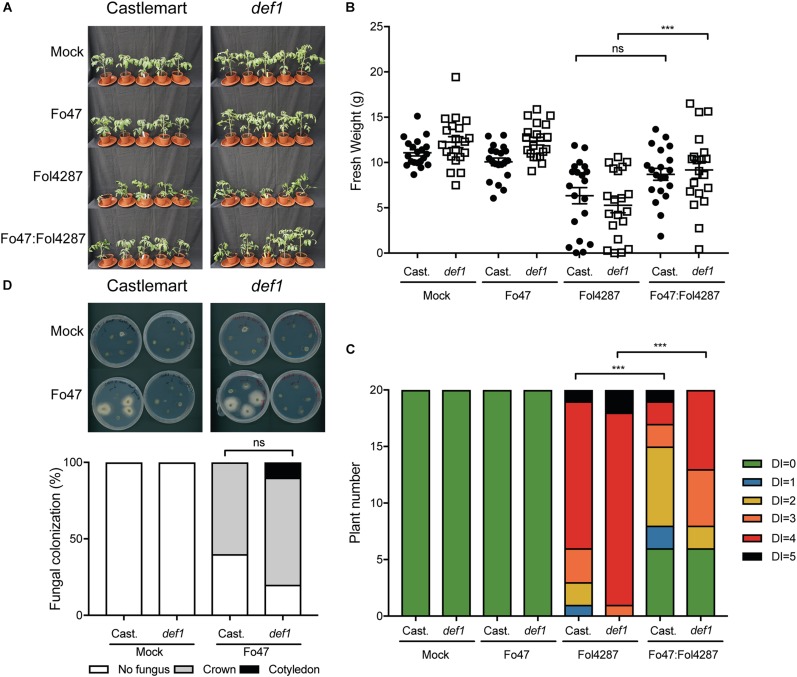
Impaired jasmonic acid biosynthesis does not affect EMR triggered by Fo47. **(A)** Ten days old tomato seedlings (Castlemart) and *def1* plants impaired in JA production were inoculated with water (mock), Fo47, Fol4287 or co-inoculated with Fo47 and Fol4287. Disease development was assessed by measuring fresh weight **(B)** and disease index **(C)** 3 weeks after inoculation. Raw data was analyzed by a non-parametric Mann–Whitney *U*-test (^*ns*^*P* > 0.05, ^∗∗∗^*P* < 0.001). The bioassay was performed three times with similar results [where the number of plants (n) was *n* = 10 for the first repetition and *n* = 20 for the second and third repetition]. **(D)** Representative stem pieces taken from crown (left) and cotyledon (right) level of five individual plants incubated for 4 days on PDA plates. Percentage of Fo47 colonization on mock-inoculated plants (*n* = 10) and Fo47 treated plants (*n* = 20). The experiment was performed twice with similar results. Raw data was analyzed by a non-parametric Mann–Whitney *U*-test (^∗∗∗^*P* < 0.001).

Beneficial soil-borne microbes have been shown to induce ISR via JA and ET, which raises the possibility that EMR could be mediated via one or both of these two plant hormones (Hossain et al., 2008; Korolev et al., 2008; Stein et al., 2008). To assess the involvement of JA in EMR we used the tomato line *def1*, which fails to accumulate JA due to a defect in the jasmonate biosynthesis pathway, for bioassays with endophytic and pathogenic Fusarium strains. Tomato plants inoculated with the pathogenic Fol4287 strain showed external Fusarium wilt disease symptoms in the Castlemart cultivar as well as in the *def1* line (Figure 3A). However, EMR was induced in both the wild-type cultivar and the JA deficient line (Figure 3A). Plants deficient in JA biosynthesis were significantly smaller when inoculated with Fol4287 than when co-inoculated with Fo47 and Fol4287 (Figure 3B). Co-inoculation of the Castlemart cultivar, however, did not result in a significantly higher fresh weight than Castlemart plants solely inoculated with the pathogenic Fol4287 isolate in two out of three experiments (Figure 3B). Fol4287-inoculated plants consistently showed vascular browning symptoms at the cotyledon level 3 weeks after inoculation of Castlemart or *def1* lines (Figure 3C). These disease symptoms were significantly reduced upon co-inoculation of the *def1* line (Figure 3C).

To determine whether plant-derived JA could influence colonization of tomato stems by Fo47, this strain was re-isolated from inoculated tomato plants. The endophyte was re-isolated from the crown level in 60% of the Castlemart plants, while in *def1* plants the endophytic strain was isolated in 85% of the cases. However, this difference in colonization was found not to be significant (Figure 3D). Overall, these data indicate that plant-derived JA does not contribute to EMR nor affects colonization by endophytic Fusarium.

### ET Biosynthesis and Perception Does Not Affect EMR

EMR against pathogens and host colonization by endophytes has been reported to be affected by ET (Kavroulakis et al., 2007). To determine whether ET plays a role in EMR, *ACD* and *Nr* tomato lines were used. The *ACD* line constitutively produces an ACC deaminase that cleaves the ET precursor 1-aminocyclopropane-1-carboxylix acid (ACC) into α-ketobutyrate and ammonium, resulting in reduced ET levels (Klee et al., 1991). The *Nr* tomato line carries a single amino acid substitution in a protein homologous to the Arabidopsis ET receptor ETR1, which results in dominant insensitivity to ET (Wilkinson et al., 1995). Fusarium bioassays on *ACD* and *Nr* tomato lines, in which respectively ET-biosynthesis or perception is impaired, were performed and Fo47 was re-isolated from the stems to monitor its colonization potential.

**FIGURE 4:**
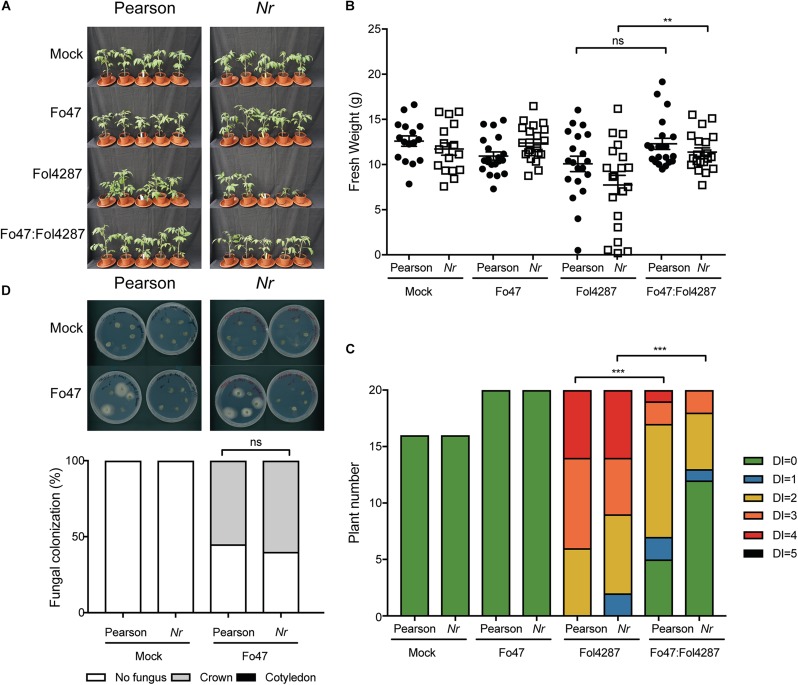
EMR is not affected in tomato plants impaired in to ethylene production. **(A)** Ten days old tomato seedlings (UC82B) and *ACD* plants impaired in ET production were inoculated with water (mock), Fo47, Fol4287 or co-inoculated with Fo47 and Fol4287. Disease development was assessed by measuring fresh weight **(B)** and disease index **(C)** 3 weeks after inoculation. Raw data was analyzed by a non-parametric Mann–Whitney *U*-test (^*ns*^*P* > 0.05, ^∗∗^*P* < 0.01, ^∗∗∗^*P* < 0.001). The bioassay was performed two times with similar results (*n* = 10 for the first repetition and *n* = 20 for the second). **(D)** Representative stem pieces taken from crown (left) and cotyledon (right) level of five individual plants incubated for 4 days on PDA plates. Percentage of Fo47 colonization on mock-inoculated plants (*n* = 10) and Fo47 treated plants (*n* = 20). The experiment was performed two times with similar results. Raw data was analyzed by a non-parametric Mann–Whitney *U*-test (^*ns*^*P* > 0.05).

As reported by Di et al. (2017), UC8B2 and *ACD* lines were susceptible to Fol4287 (Figure 4A). When co-inoculated with Fo47 and Fol4287 the tomato plants displayed only occasionally mild disease symptoms (Figure 4A). Fol4287-inoculated UC82B and *ACD* plants showed a reduction in fresh weight as compared to mock treatment (Figure 4B). Co-inoculated plants showed higher fresh weight (Figure 4B), and have fewer disease symptoms (Figure 4C) as compared to Fol4287-inoculated plants.

**FIGURE 5:**
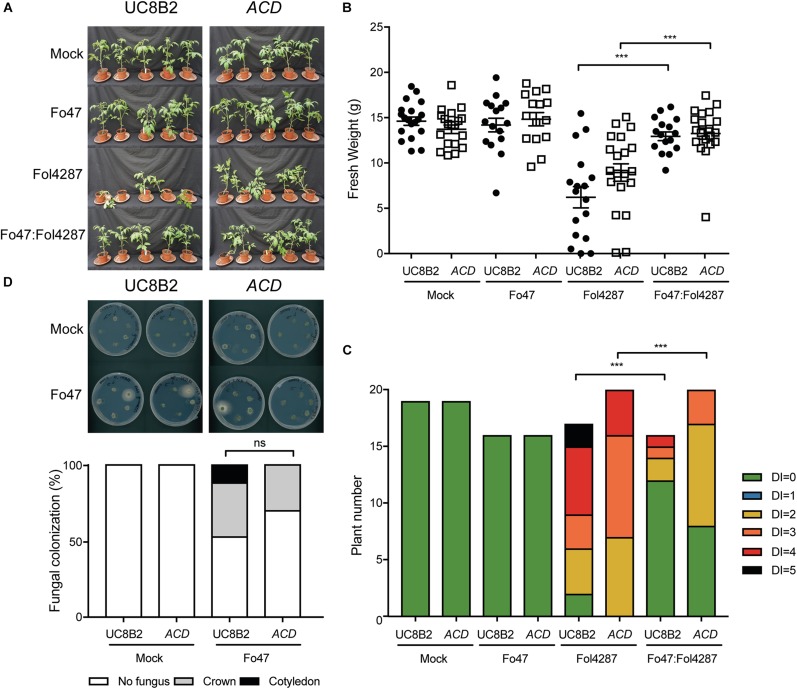
Impaired ethylene perception in tomato does not affect EMR triggered by Fo47. **(A)** Ten days old wild-type tomato seedlings (Pearson) and *Nr* plants impaired in ET perception were inoculated with water (mock), Fo47, Fol4287 or co-inoculated with Fo47 and Fol4287. Disease development was assessed by measuring fresh weight **(B)** and disease index **(C)** 3 weeks after inoculation. Raw data was analyzed by a non-parametric Mann–Whitney *U*-test (^∗∗∗^*P* < 0.001). The bioassay was performed twice with similar results [where the number of plants (n) was *n* = 10 for the first repetition and *n* = 20 for the second repetition]. **(D)** Representative stem pieces taken from crown (left) and cotyledon (right) level of five individual plants incubated for 4 days on PDA plates. Percentage of Fo47 colonization on mock-inoculated plants (*n* = 10) and Fo47 treated plants (*n* = 20). The experiment was performed twice with similar results. Raw data was analyzed by a non-parametric Mann–Whitney *U*-test (^∗∗∗^*P* < 0.001).

To investigate whether ET affects host colonization by the endophyte, stem pieces from Fo47-treated plants were collected to re-isolate the fungus. No statistical difference could be observed in Fo47 colonization between the wild-type line and the line *ACD* (Figure 4D). Despite the inability of *ACD* plants to produce ET, it is possible that these plants could still be exposed to ET since Fol has been shown to produce ET (Gentile and Matta, 1975). Therefore, we performed the same bioassays as described above with a *Nr* tomato line, which is insensitive to ET, as well as the parental Pearson line. The Pearson cultivar showed fewer disease symptoms upon Fol inoculation than the other lines tested in this study (Figure 5A) and has a fresh weight similar as the mock treatment (Figure 5B). The *Nr* line was susceptible to Fol4287 showing a clear reduction in fresh weight as compared to the mock treatment (Figure 5B). Plants co-inoculated with the pathogenic and the endophytic strain did not display strong disease symptoms (Figure 5A). Co-inoculated *Nr* plants showed a significant increase in fresh weight when compared with Fol4287, but this was not the case in the Pearson wild-type line (Figure 5B). Despite the fact that *Nr* and Pearson Fol4287-inoculated plants did not show a clear size reduction in both tomato lines, these lines were susceptible to Fol4287 infection and developed brown vessels indicative of disease (Figure 5C). Moreover, disease symptoms were reduced in co-inoculation treatments as compared with Fol4287-inoculated plants in *Nr* plants, suggesting that ET perception is not involved in EMR.

To assess whether ET sensing may be necessary for endophytic colonization, Fo47 was re-isolated from stems. The endophytic strain Fo47 was found to colonize stems of wild-type and *Nr* tomato to a similar extent, indicating that plant ET sensing is also not involved in mediating endophytic colonization of tomato (Figure 5D). In summary, these data suggest that ET is not involved in EMR nor plays a role in colonization by a Fusarium endophyte.

### Callose Deposition in Tomato Roots Appears Unaffected by Fungal Colonization

**FIGURE 6:**
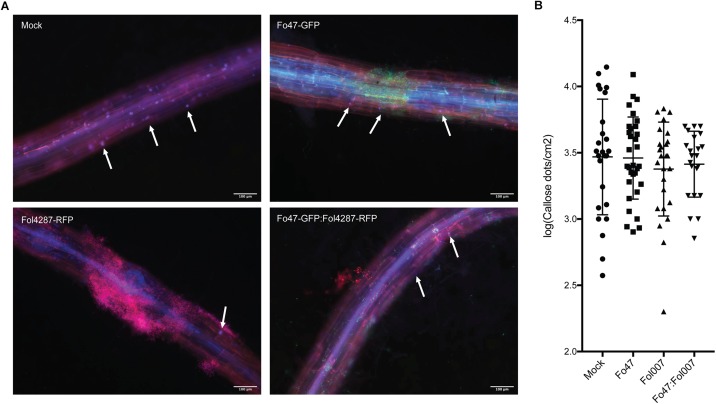
Callose deposition in response to fungal colonization. **(A)** Ten days old C-32 tomato seedlings were inoculated with water (mock), Fo47-GFP, Fol4287-RFP or co-inoculated with Fo47-GFP and Fol4287-RFP. Three days after inoculation tomato roots were stained with aniline blue, and callose depositions were analyzed using a Digital Inverted Fluorescence EVOS Microscope (magnification 10×). Scale bar = 100 μm. Arrow heads point to callose depositions. **(B)** Ten days old C-32 tomato seedlings were inoculated with water (mock), Fo47-RFP, Fol007-GFP or co-inoculated. Quantification of the number of callose dots per root area as determined using the Image J Fiji plugin. A minimum four plants per treatment were analyzed using at least three pictures per plant. Data was normalized by log transformation and for statistical analysis an ANOVA test was performed. The experiment was performed thrice with similar results.

To assess if callose depositions could be involved in EMR, 10 days old tomato seedlings were inoculated with water (mock), and Fo47 and/or Fol race 2 (Fol4287 or Fol007). To aid detection of the fungus in the roots stably transformed strains were used that constitutively express either red or green fluorescent protein; Fo47 and Fol. Three days after inoculation roots were treated with aniline blue to stain for callose accumulation. Subsequently, the stained callose and fungal colonization were visualized by fluorescence microcopy. Distinct blue fluorescent dots (callose) were observed across all treatments and specifically at root tips and at damage sites (data not shown). As callose deposition at root tips appeared to be a normal plant response, pictures from root tips were excluded from further analysis, However, the damaged sites could not be excluded from our analysis since Fusarium uses these wounds as entry points of the root. Therefore, it was not possible to distinguish whether the callose deposited at damaged sites is due to fungal colonization, or results from the plant damage response. Callose depositions were detected in all four treatments both near fungal mycelium and at distant sites (Figure 6). Quantification of the number of callose depositions per area did not reveal significant differences between mock, Fo47, Fol or the co-inoculation treatment. It is noteworthy to point out that one plant can have roots that show few to no callose depositions (Supplementary Figure S2 Mock^∗^) while other roots show a high number of callose depositions (Supplementary Figure S1 Mock). This high variability is also reflected by the wide distribution of the data points, and the concomitant high standard deviation in the graph (Figure 6) when plotting the results.

In conclusion, no significant differences in callose depositions were observed in response to inoculation with pathogen, endophyte or both.

## Discussion

In this study, we demonstrate that Fo47 can confer EMR independently of SA, ET, or JA – the major hormones that have commonly been associated with ISR or SAR (Pieterse et al., 2014). EMR was successfully established in (transgenic) tomato lines defective in SA accumulation, ET-production or -perception and JA biosynthesis. Our findings imply that EMR is mechanistically distinct from ISR and SAR. In our experiments, equal concentrations of Fo47 and Fol4287 were co-inoculated. A typical SAR/ISR response is based on initial exposure to either an avirulent pathogen or to an endophyte that primes plant defense and a subsequent infection with the pathogen. The prompt resistance observed in our studies implies that other mechanisms are involved, such as antibiosis, competition for niches or nutrients, or a plant response independent from SA, ET and JA. Since Fo47, and other non-pathogenic Fusarium strains, also reduce Fol disease symptoms in a split-root system (Kroon et al., 1991; Fuchs et al., 1997), we speculate that Fo47 at least partially confers resistance via the plant.

Transgenic *NahG* plants were still able to mount an EMR response, conferring resistance to Fusarium wilt when exposed to Fo47. This finding was unexpected, as EMR studies in the Solanaceae species pepper (*Capsicum annuum*) implied involvement of this phytohormone in induced immunity (Veloso et al., 2016). Pepper roots inoculated with Fo47 accumulated higher SA levels than non-inoculated plants (Veloso et al., 2016). Furthermore, also in tomato roots and cotyledons Fo47 infection increased expression of the SA-marker gene *PR1a* (Aimé et al., 2013). Based on the ability of Fo47 to potentiate expression of SA-defense marker genes the endophyte was proposed to confer protection against Fol by priming SA-mediated defenses (Aimé et al., 2013). Our data, and that by Di and co-workers, confirm that SA is important for susceptibility to Fusarium infection (Di et al., 2017) as both studies showed induction of *PR1a* upon Fol4287 inoculation (Figure 1A). However, although SA marker genes are induced in the interaction between plant and pathogen, our data show that these genes are not induced following colonization by the Fo47 endophyte, nor upon co-inoculation of Fo47 with the pathogen. RT-PCR data shows that *ICS* and *PAL*, two genes involved in SA biosynthesis, were not consistently upregulated upon co-inoculation (Supplementary Figure S1C), although *PAL* expression was slightly induced in the second experiment (Supplementary Figure S1C). Furthermore, 3 weeks after Fo47 colonization no induction of *PR1a* was observed, implying that SA signaling is either not or only transiently induced during inoculation. In line with this, we did not observe induction of the *PR1a* gene upon co-inoculation. Our results are in accordance with xylem sap proteomic studies in which PR1a was found to be significantly induced only upon colonization by a pathogenic strain, not by an endophyte or during EMR (de Lamo et al., 2018). Of note, it has been reported that EMR triggered by the bacterium *Paenibacillus alvei* K165 in *Arabidopsis* to *Verticillium dahliae* was unaffected in *NahG* plants but was compromised in *npr1*, *sid2, or eds5/sid1* plants (Tjamos et al., 2005). These observations imply that SA (signaling) itself is not required for EMR, but an intact SA biosynthesis pathway is. A similar situation could apply for Fo47-triggered resistance. In future experiments tomato SA biosynthesis mutants could be generated and their ability to mount EMR assessed to put this hypothesis to the test. In conclusion, our findings indicate that SA accumulation and signaling are not involved, or only have a marginal role, in EMR.

Results from Fusarium bioassays on plants impaired in ET biosynthesis (*ACD*) or ET sensing (*Nr*) imply that EMR is not mediated via ET. In our set-up, Fo47-mediated resistance against Fol was apparent by a reduction in leaf epinasty and/or yellowing symptoms in comparison to the diseased controls. ET aggravates disease symptoms in susceptible tomato plants inoculated with Fol strains (Gentile and Matta, 1975; Di et al., 2017), and ET can be produced by Fol (Gentile and Matta, 1975). Tomato mutants that are unable to produce or perceive ET indeed appeared to develop less severe disease symptoms as the wild-type Pearson controls, although the differences were not statistically significant in our experiments (Figure 5). Three weeks after Fol4287 infection, expression of the *ethylene receptor* (*ETR4*) was not induced, but expression of *Pti4*, an ethylene responsive factor, was. The degree of induction (two-fold) was rather modest as compared to that reported in previous reports (10-fold) (Di et al., 2017), and a difference in expression was also not consistently observed in our assays (Supplementary Figure S1E). One explanation for the differences between both studies could be the genetic background of the plants used (UC8B2 versus C32) and different time points of sampling (2 versus 3 weeks post-inoculation). Notably, neither of these markers was induced upon inoculation of Fo47, either alone or in combination with Fo4287. These findings show that ET signaling is not induced by the endophyte or during EMR and that the hormone is merely involved in disease symptom development upon Fol infection.

Use of the *Nr* mutant line excluded the possibility that ET-dependent signaling pathways are activated by fungus-produced ET. Since Fo47 mounts EMR in both *Nr* and *ACD* plants it can be concluded that neither ET production nor sensing/signaling is required for this induced resistance. Our findings that EMR was not altered in *Nr* mutant plants contrasts a study with an endophytic *Fusarium solani* isolate, Fs-K. Fs-K was reported to confer protection against *F. oxysporum* f. sp. *radicis-lycopersici* (Forl) in tomato plants (Kavroulakis et al., 2007). When inoculating *Nr* mutants, or *epi* mutants that exert increased ET synthesis and ET responses (Barry et al., 2001), no Fs-K-mediated EMR was observed (Kavroulakis et al., 2007). In addition, when co-inoculating Fs-K and Forl in 1:1 ratio, the resistance conferred by FS-K did not occur and the plants became diseased (Kavroulakis et al., 2007). This is a relevant observation, as it suggests that Fo47 triggers EMR differently than FS-K, as we did still observe EMR when using a 1:1 ratio of endophyte and pathogen. So, either there are distinct types of EMR that are induced by endophytic *F. oxysporum* and *F. solani* strains, or the EMR against Fusarium wilt disease is different from the one controlling Fusarium root-rot disease.

Fusarium bioassays on the *def1* mutant, which is incapable of producing JA, revealed that JA does not play a major role in EMR, which agrees with the observed unaltered expression of the JA marker gene *PI-I*. It has been reported that JA is not involved in Fol-mediated disease development in 6 weeks old tomato (Di et al., 2017). In our set-up, we observe that compromised JA signaling marginally increased pathogenicity of Fol in seedlings, which could suggest a minor role of JA in younger plantlets (Figure 2). This increase in pathogenicity would also explain the less efficient containment of the pathogen by EMR upon co-inoculation of the *def1* mutant. Fo47-infected pepper roots transiently accumulate JA-Ile at 48 h post-inoculation, and this increase disappears after 72 h, implying a transient role of the hormone early in host colonization (Veloso et al., 2016). An Arabidopsis-infecting Fusarium strain (*Fusarium oxysporum* f. sp. *conglutinans*) has been show to produce jasmonates and these promote infection (Cole et al., 2014). However, in axenic Fol4287 cultures no jasmonates were detected, suggesting that Fol does not produce this phytohormone (Cole et al., 2014). It is currently unknown how fungi produce jasmonates (Oliw and Hamberg, 2017), or whether Fo47 can produce them. Hence, at this stage we cannot exclude the possibility that Fo47 secretes JA – or JA derivatives – that are involved in EMR. Unfortunately, no JA insensitive mutants in tomato are available to explore this possibility in bioassays. Nevertheless, our data show that plant-derived JA is not required for EMR in tomato and together with the observation that *PI-I* expression is not induced supports the hypothesis that JA signaling is not activated in tomato during Fol infection or during EMR. SA and JA act antagonistically in tomato in susceptibly to Fol (Robert-Seilaniantz et al., 2011), however since in EMR neither SA nor JA signaling appear to be induced, crosstalk is not anticipated to be involved in this response.

Our re-isolation experiments revealed that Fo47 is often able to colonize the crown of inoculated tomato plants. Host colonization appears to be restricted to the crown level as we very rarely observed colonization at the cotyledon level. The Fo47 colonization pattern in stems of tomato lines impaired in SA accumulation, ET-production or -sensing or in JA biosynthesis was similar to that of wild-type plants. It was unexpected that SA, ET and JA do not affect colonization of the stem by endophytic Fusarium, as these hormones strongly affect stem colonization by pathogenic Fol strains (Di et al., 2017). Our observation that Fo47 did not induce expression of any of the analyzed SA-, JA-, or ET- markers is consistent with the insignificance of these hormones for Fo47 colonization. Apparently, host colonization by the endophyte or pathogen is controlled by distinct mechanisms in the plant. This idea is consistent with the different colonization patterns observed for each strain. Whereas the endophyte typically colonizes cortex and epidermal tissues of the root, the pathogen favors endodermis and vasculature (Alabouvette et al., 2009).

Fo47 inoculation has been shown to induce cell wall appositions and/or papillae formation in the invaded epidermis and outer cortex, and in non-invaded xylem vessels of pea roots, which correlates with the restricted Fo47 colonization of the outermost root tissues (Benhamou and Garand, 2001). Fo47-triggered formation of papilla, containing callose and lignin deposits, could represent a mechanism to impede Fol colonization. A less efficient root colonization is predicted to result in a reduced ability to reach the xylem vessels and hence a reduction in disease symptoms. To assess whether Fo47 triggers a similar fortification response in tomato roots as in pea we stained the roots with aniline blue and monitored callose depositions. Callose deposits were visible as distinct blue dots along the root vasculature, showing a higher abundance near the root tip and at damaged root parts (irrespective of the treatment). No obvious differences could be observed in abundance, intensity or distribution of callose dots between the different treatments. As we did not perform time lapse experiments we cannot exclude occurrence of early differences as reported before (Beckman et al., 1982). Despite not observing differences in callose depositions during EMR, cell wall fortification is likely to be involved in EMR. A recent proteomic study of tomato plants co-inoculated with Fo47 and Fol showed accumulation of proteins associated with lignification, which support this idea (de Lamo et al., 2018). Furthermore, in line with the notion that an intact SA biosynthesis pathway is required for bacterial induced EMR in Arabidopsis (Tjamos et al., 2005), it is tempting to speculate that it is not the signaling function of SA that is required for the induced resistance phenotype, but the benzoate intermediates that it provides (Dempsey et al., 2011). These compounds are the major precursors for lignification and production of antimicrobial compounds in plants (Dempsey et al., 2011). Analyzing the lignin content and content of (poly)phenolic compounds in inoculated roots following EMR induction could reveal whether there is a correlation between these two events, providing a mechanistic basis for the observed resistance.

Induction of lignification and/or cell wall fortifications restricting access to the root vasculature could provide an explanation why protection by Fo47 is especially effective against wilt causing fungi such as Fol and *Verticillium dahliae.* A mechanical barrier that specifically prevents access to the vasculature would also explain why EMR is ineffective in conferring resistance against foliar pathogens such as *Botrytis cinerea* or *Phytophthora capsici* (Veloso et al., 2016). A similar induced resistance mechanism might also operate in other plant species and even in stems, as exemplified by the observation that injection of non-pathogenic *Verticillium* strains in the vasculature of Elm trees confers protection against the causal agent of Dutch Elm disease, *Ophiostoma ulmi* (Scheffer, 1990). Together, our findings suggest that, besides ISR and SAR, an additional inducible defense mechanism exists in plants that confers protection against vascular pathogens. It will be interesting to examine whether EMR also controls bacterial wilt diseases, like those caused by the bacteria *Xylella fastidiosa* or *Xanthomonas campestris*. Unraveling the molecular mechanism of this type of resistance holds potential for improved control of wilt diseases without compromising, or affecting, the conventional defense pathways that are mediated by SA, ET or JA.

## Author Contributions

MC, FT, and MR designed the experiments. MC and FdL performed the bioassays including the fungal re-isolation experiments. MC performed the RNA extractions and RT-qPCR experiments. MC and BV performed the callose staining experiments. FdL, BV, FT, and MR, gave intellectual input and critically revised the manuscript. MC and FT wrote the manuscript.

## Conflict of Interest Statement

The authors declare that the research was conducted in the absence of any commercial or financial relationships that could be construed as a potential conflict of interest.

## Footnotes

1 https://www.graphpad.com

## Supplementary Material

FIGURE S1 Expression of SA, JA or ET marker genes is unaffected by EMR. **(A)** Ten days old wild-type tomato seedlings (C32) inoculated with water (mock), Fo47, Fol4287 or co-inoculated with Fo47 and Fol4287. **(B)** Disease development as assessed by measuring fresh weight and disease index 3 weeks after inoculation. Raw data was analyzed by a non-parametric Mann–Whitney *U*-test (ns *P* > 0.05, ^*^*P* < 0.05). **(C)** Expression levels of the SA biosynthesis genes *isochorismate synthase* (*ICS*) and *phenylalanine ammonia-lyase* (*PAL*) or the *pathogenesis-related 1a* (*PR1a*) SA maker gene in Fo47, Fol4287 or in co-inoculated plants. **(D)** Expression levels of the JA reporter gene *proteinase inhibitor* (*PI-I*) in Fo47, Fol4287 or in co-inoculated plants. **(E)** Expression levels of the ET-regulated marker genes *ethylene responsive factor* (*Pti4*) and *ethylene receptor* (*ETR4*) in Fo47, Fol4287 or in co-inoculated plants. Gene expression levels were measured using RT-qPCR and depicted relative to that of *tubulin*. Five biological replicates per each treatment were analyzed. The different letters represent a significant difference at *P* < 0.05 as determined by ordinary one-way ANOVA with Tukey’s multiple comparisons test.

FIGURE S2 Representative pictures showing callose deposition in mock, Fo47-GFP, Fol4287-RFP or co-inoculated tomato roots. Pictures were taken using the Digital Inverted Fluorescence EVOS Microscope (magnification 10×) using the bright field, DAPI filter for visualizing callose depositions, GFP filter for visualizing GFP-labeled Fo47, and Texas Red filter for visualizing RFP-labeled Fol4287. Merged pictures were obtained by composing the image in Fiji (Image J), of the blue (DAPI), red (Texas Red), and green (GFP) channels. Scale bar = 100 μm. Mock^∗^ depicts an example of a root that has only few callose depositions, while the Mock panel below shows another root from the same plant with a high number of depositions.

TABLE S1 Primer sequences used for RT-qPCR analysis.
