# Supplementary figures and images for: Endophyte-Mediated Resistance in Tomato to Fusarium oxysporum Is Independent of ET, JA, and SA
Source: Front Plant Sci. 2019 Jul 31;10:979. doi: 10.3389/fpls.2019.00979 (PMC6685397; doi:10.3389/fpls.2019.00979)

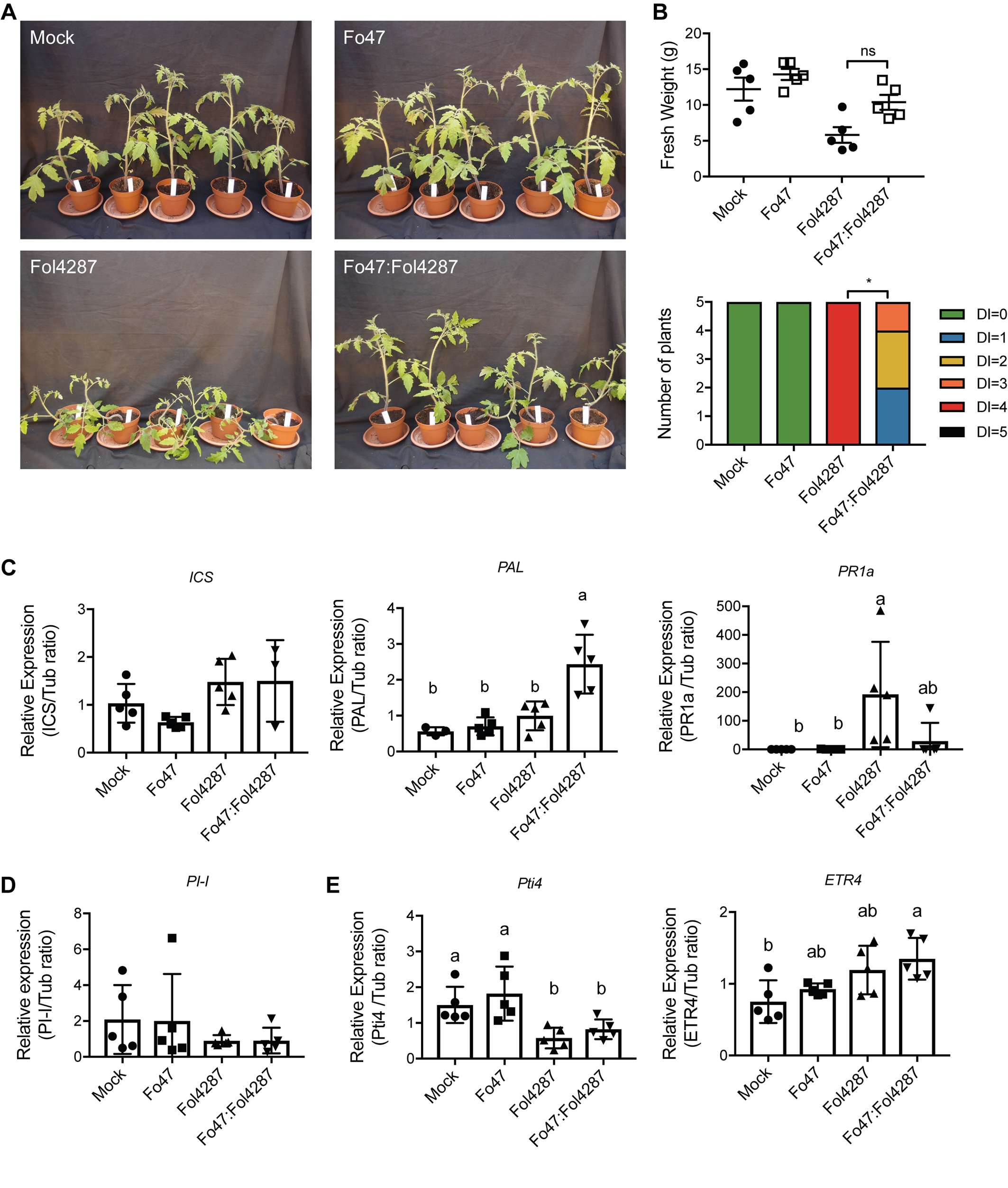

Supplement: FIGURE S1 — Expression of SA, JA or ET marker genes is unaffected by EMR. (A) Ten days old wild-type tomato seedlings (C32) inoculated with water (mock), Fo47, Fol4287 or co-inoculated with Fo47 and Fol4287. (B) Disease development as assessed by measuring fresh weight and disease index 3 weeks after inoculation. Raw data was analyzed by a non-parametric Mann–Whitney U-test (ns P > 0.05, *P < 0.05). (C) Expression levels of the SA biosynthesis genes isochorismate synthase (ICS) and phenylalanine ammonia-lyase (PAL) or the pathogenesis-related 1a (PR1a) SA maker gene in Fo47, Fol4287 or in co-inoculated plants. (D) Expression levels of the JA reporter gene proteinase inhibitor (PI-I) in Fo47, Fol4287 or in co-inoculated plants. (E) Expression levels of the ET-regulated marker genes ethylene responsive factor (Pti4) and ethylene receptor (ETR4) in Fo47, Fol4287 or in co-inoculated plants. Gene expression levels were measured using RT-qPCR and depicted relative to that of tubulin. Five biological replicates per each treatment were analyzed. The different letters represent a significant difference at P < 0.05 as determined by ordinary one-way ANOVA with Tukey’s multiple comparisons test. [file Image_1.TIF]

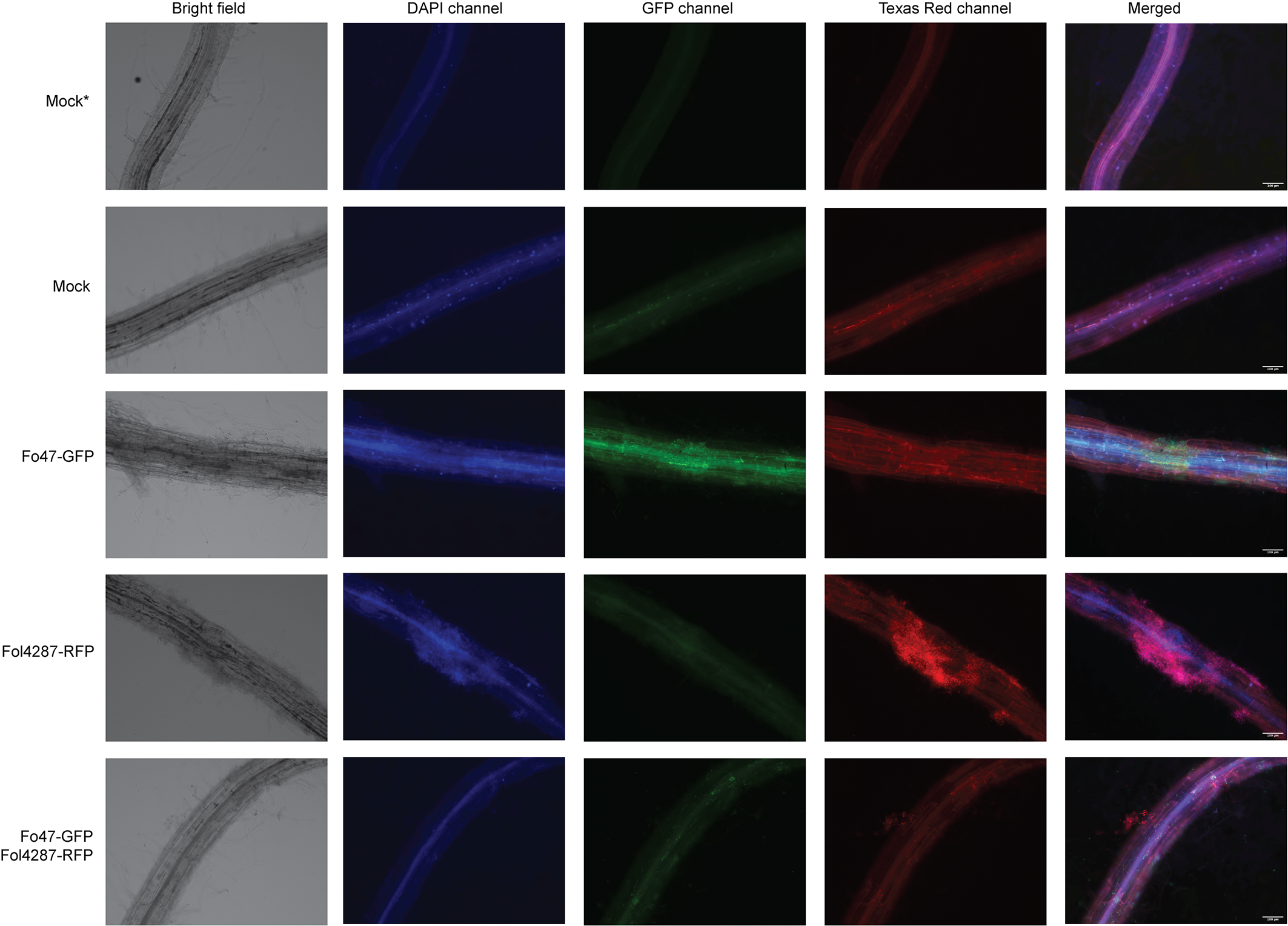

Supplement: FIGURE S2 — Representative pictures showing callose deposition in mock, Fo47-GFP, Fol4287-RFP or co-inoculated tomato roots. Pictures were taken using the Digital Inverted Fluorescence EVOS Microscope (magnification 10×) using the bright field, DAPI filter for visualizing callose depositions, GFP filter for visualizing GFP-labeled Fo47, and Texas Red filter for visualizing RFP-labeled Fol4287. Merged pictures were obtained by composing the image in Fiji (Image J), of the blue (DAPI), red (Texas Red), and green (GFP) channels. Scale bar = 100 μm. Mock∗ depicts an example of a root that has only few callose depositions, while the Mock panel below shows another root from the same plant with a high number of depositions. [file Image_2.TIF]
