# Supplementary material for: Endophyte-Mediated Resistance in Tomato to Fusarium oxysporum Is Independent of ET, JA, and SA
Source: Front Plant Sci. 2019 Jul 31;10:979. doi: 10.3389/fpls.2019.00979 (PMC6685397; doi:10.3389/fpls.2019.00979)
Supplement: TABLE S1 — Primer sequences used for RT-qPCR analysis. [file Table_1.docx]

**Table S1 | Primer sequences used for RT-qPCR analysis.**

| Gene | Primer names | Forward primer (5’-3’) | Reverse primer (5’-3’) |
| --- | --- | --- | --- |
| α-tubulin | FP:2149; FP:2150 | TCGTGGCCACTATACCATTG | AGTGACCCAAGACCTGAACC |
| PR1a | FP:6732; FP:6733 | TGGTGGTTCATTTCTTGCAACTAC | ATCAATCCGATCCACTTATCATTTTA |
| Pti4 | FP:6280; FP6281 | TCGTCGGGAAACGGTTCCAT | GACATCCAACTTGCATGACACTTG |
| ETR4 | FP:6795; FP:6796 | GGTAATCCCAAATCCAGAAGGTTT | CAATTGATGGCCGCAGTTG |
| PAL | FP:5056; FP:5057 | CGTTATGCTCTCCGAACATC | GAAGTTGCCACCATGTAAGG |
| PI-I | FP:5864; FP:5865 | GTGTACCAACAAAGCTTGCTAAAGA | GTACAACAACACCCAAAATGTTGTC |
| ICS | FP:7620; FP:7621 | TCCAGGCTGAAGATGATGAG | TTATTCCAACCGCAAATTCA |
